# Does pisiform subluxation affect the postoperative outcomes in a cohort of patients with distal radius fractures?

**DOI:** 10.1016/j.amsu.2018.09.028

**Published:** 2018-09-25

**Authors:** Yoichi Sugiyama, Kiyohito Naito, Hiroyuki Obata, Mayuko Kinoshita, Kenji Goto, Nana Nagura, Yoshiyuki Iwase, Kazuo Kaneko

**Affiliations:** a Department of Orthopaedics, Juntendo University School of Medicine, 2-1-1 Hongo, Bunkyo-ku, Tokyo, 113-8421, Japan; b Department of Orthopaedic Surgery, Juntendo Tokyo Koto Geriatric Medical Center, 3-3-20 Shinsuna, Koto-ku, Tokyo, 136-0075, Japan

**Keywords:** Computed tomography, Concomitant injuries, Distal radius fractures, Pisiform subluxation, Pisotriquetral joint

## Abstract

**Background:**

In this study, we retrospectively surveyed the presence or absence of pisiform subluxation in surgically treated distal radius fractures (DRF) cases. In addition, we investigated whether or not the postoperative short-term treatment outcome differs due to the presence of pisiform subluxation.

**Materials and methods:**

The subjects were 134 DRF patients treated with volar locking plate fixation (53 males and 81 females, mean age: 64 years old). The pisotriquetral joint was observed on a preoperative CT to investigate the presence or absence of pisiform subluxation according to the criteria reported by Vasilas. 134 patients divided into subluxation group and non-subluxation group, and the clinical outcomes were compared between these groups.

**Results:**

Pisiform subluxation was noted in 23.1% (31 patients, 15 males and 16 females, mean age 61 years). No significant difference was noted in patient background in both groups. The postoperative pronation angle in the non-subluxation group was significantly greater than that in the subluxation group, but there was no significant difference in any other parameter (the range of motion of the wrist, grip strengths, VAS, Q-DASH scores, and Mayo score) between these 2 groups. However it concomitantly occurred in 23.1% of DRF cases in our series, there was no significant difference in the postoperative treatment outcome between these 2 groups.

**Conclusions:**

Therapeutic intervention of pisiform subluxation is unnecessary during treatment of DRF, since pisiform subluxation does not affect the postoperative clinical outcomes of distal radius fractures.

## Highlights

• Pisiform subluxation occurred in 23.1% of distal radius fracture cases, suggesting that it is not a rare pathology.
• Therapeutic intervention of pisiform subluxation may be unnecessary during treatment of distal radius fracture.
• The therapeutic intervention of pisotriquetral joint disorders should be considered when they develop.

## 1 Introduction

Various traumas concomitantly occur around the carpal bones with distal radius fractures (DRF) [[1], [2], [3]], but some traumas may be overlooked on examination by plain radiography alone [2]. DRF are common traumas, examined not only by hand surgeons but also orthopedists. Imaging using computed tomography (CT) as well as plain radiography has become generally used to judge the fracture type [4], with which collection of detailed information on not only distal radius fractures but also fractures and subluxation of the carpal bone has become possible.

Case reports of pisiform subluxation and DRF-complicating pisiform subluxation have occasionally been reported, but there is no standardized treatment strategy [[5], [6], [7]]. Regarding the diagnostic criteria of pisiform subluxation, only an old report by Vasilas et al. published in 1960 is still available [8], and many points remain unclear with regard to the pathology.

In this study, we retrospectively surveyed the presence or absence of pisiform subluxation in surgically treated DRF cases. In addition, we investigated whether or not the postoperative short-term treatment outcome differs due to the presence of pisiform subluxation.

## 2 Materials and methods

The study was approved by the Ethics Committee for Medical Research of our University. Informed consent was obtained from all individual participants included in the study.

One hundred seventy-three displaced distal radius fractures patients who underwent volar locking plate fixation between September 2012 to June 2015 and follow up with X-rays and clinical outcomes at three months were included as part of the study. The study comprised of 134 patients (53 males and 81 females, mean age: 64 years old). All patients were surgically treated with volar locking plate fixation. The pisotriquetral joint was observed in the sagittal view on a preoperative CT to investigate the presence or absence of pisiform subluxation. Pisiform subluxation was diagnosed according to the criteria reported by Vasilas [8]: conditions meeting 4-mm or more dilatation of the joint space, or 2-mm or more dislocation of the pisiform joint surface toward the distal or proximal side. Then 134 patients divided into subluxation group and non-subluxation group. Both groups were compared with regard to their background (age, gender, fracture type, and direction of fracture dislocation) as well as their outcome three months postoperatively (wrist motion, grip strength (%ratio relative to that on the healthy side), Visual Analog Scale (VAS), Quick Disabilities of the Arm, Shoulder and Hand (Q-DASH) score, Mayo wrist score).

Data are expressed as mean ± SD. Statistic significant differences of patient's backgrounds was analyzed by the Fishers exact test and that of postoperative outcomes (wrist motion, grip strength, VAS, Q-DASH score, and Mayo wrist score) was analyzed by the Mann-Whitney U tests (Prism 4, GraphPad Software, San Diego, CA). Differences were considered statistically significant at P < 0.05.

## 3 Results

Pisiform subluxation was noted in 23.1% (31 patients (15 males and 16 females), mean age: 61 years old), and the fracture type based on the Arbeitsgemeinschaft für Osteosynthesefragen (AO) classification [9] was A2 in 10 patients, A3 in 1, B2 in 1, B3 in 2, C1 in 10, C2 in 1, and C3 in 6. The fracture was dislocated toward the dorsal side in 24 patients and palmar side in 7. Pisiform subluxation was absent in 76.9% (103 patients (38 males and 65 females), mean age: 64 years old), and the AO classification fracture type was A2 in 29 patients, A3 in 2, B2 in 1, B3 in 3, C1 in 44, C2 in 8, and C3 in 16. The fracture was dislocated toward the dorsal side in 89 patients and palmar side in 14. No significant difference was noted in patient background (Table 1).

**Table 1:** Patient backgrounds of the subluxation and non-subluxation groups.

	Subluxation groups (n = 31)	Non-subluxation groups (n = 103)	Statistical analysis
Sex (M: F)	15:16	38: 65	N.S.
Age	61 ± 18	64 ± 15	N.S.
AO classification	Type A: 11	Type A: 31	N.S.
	Type B: 3	Type B: 4	
	Type C: 17	Type C: 68	
Displacement	Dorsal: 24	Dorsal: 89	N.S.
	Volar: 7	Volar: 14	

M: male, F: female, N.S.: not significant.

The range of motion of the wrist joint at 3 months after surgery was 68 ± 13° on flexion, 63 ± 21° on extension, 77 ± 17° on pronation, and 88 ± 7° on supination in the subluxation group, and 67 ± 14°, 65 ± 14°, 85 ± 10°, and 83 ± 13°, respectively, in the non-subluxation group. The grip strengths (%ratio relative to that on the healthy side) were 64.6 and 64.1%, the VAS scales were 1.7 ± 1.5 and 1.7 ± 1.8, the Q-DASH scores were 16.7 ± 19.6 and 17.1 ± 18.0, and The Mayo score was excellent in 26 patients, and good in 5, and excellent in 75, good in 24, and fair in 4, in the subluxation and non-subluxation groups, respectively. Wrist pain was very few in our subjects at 3 months after surgery (VAS 1.7 or 1.8). This survey was conducted at rest. In our postoperative therapy, return to daily living behavior and operated limb load were permitted according to the pain within manageable bounds. The postoperative pronation and supination angle in the non-subluxation group was significantly greater than that in the subluxation group. On the other hand, there was no significant difference in any other parameter between these 2 groups (Table 2). No soft tissue (flexor/extensor tendons, vessels, and nerves) complications were observed in any patient.

**Table 2:** Postoperative outcomes at 3 months after surgery in the subluxation and non-subluxation groups.

		Subluxation groups (n = 31)	Non-subluxation groups (n = 103)	Statistical analysis
ROM	F	68 ± 13	67 ± 14	N.S.
	E	63 ± 21	65 ± 14	N.S.
	P	77 ± 17	85 ± 10	p < 0.05
	S	88 ± 7	83 ± 13	p < 0.05
Grasp	% of healthy side	64.6 ± 24	64.6 ± 24	N.S.
VAS		1.7 ± 1.5	1.7 ± 1.8	N.S.
Q-DASH		16.7 ± 19.6	17.1 ± 18.0	N.S.
Mayo score		81 ± 10	86 ± 13	N.S.
		Excellent: 26	Excellent: 75	
		Good: 5	Good: 24	
		Fair: 0	Fair: 4	

ROM: range of motion, F: flexion, E: extension, P: pronation, S: supination, VAS: Visual Analog Scale, Q-DASH: Quick Disabilities of the Arm, Shoulder and Hand, N.S.: not significant.

## 4 Discussion

DRF-complicating traumas around the wrist joint, such as carpal bone fractures and ligament injuries between carpal bones, cannot be diagnosed by plain radiography alone in some cases [2,10,11]. The important thing is to evaluate the presence or absence of these concomitant injuries before surgery, for which examination using CT is useful to make a surgery plan for DRF as well as evaluate concomitant injuries. Komura et al. reported that concomitant carpal bone fractures undetectable by radiography were identified by CT in 11% of cases [2].

Pisiform subluxation accompanying DRF is a complication often overlooked. Actually, pisiform subluxation was not diagnosed before surgery in any of the 134 patients. Moreover, pisiform subluxation concomitantly occurred in 23.1% of DRF cases, suggesting that it is not a rare pathology. Induction of ulnar-side pain by pisotriquetral joint injury has been reported [12], but the VAS scale was 1.7 in both pisiform subluxation and non-subluxation groups, showing no significant difference between the 2 groups, and the pain did not interfere with daily life in any patient. Then, a question arises: ‘should pisiform subluxation be regarded as a pathological condition requiring treatment?’ In the present study, there was a significant difference between the two groups in the postoperative pronation and supination angles. It is known that in supination position, the pisiform is towed by the transverse carpal ligament to ulnar side by about 3 mm. By this mechanism, we think that it is the cause of the significant difference between the two groups at the pronation and the supination angles. However, there was no significant difference in the postoperative treatment outcome between the subluxation and non-subluxation groups, suggesting that pisiform subluxation has no short-term influence on DRF.

Moojen et al. analyzed pisiform movement using CT [13]. They clarified that the pisiform moves toward the distal side and comes close to the distal triquetrum when the wrist joint is extended, whereas it moves toward the proximal side, dilating the joint space of the pisotriquetral joint, when the wrist joint is flexed, showing that the pisiform in the pisotriquetral joint is rich in mobility. Yamaguchi et al. reported that non-traumatic arthropathic changes were observed in the pisotriquetral joint in 83% of autopsied cadavers [14]. Moojen and Yamaguchi also reported that pain derived from the pisotriquetral joint is not rare, suggesting that it is a disorder due to the high mobility of the pisiform [13,14].

Leaving pisiform subluxation untreated may promote progression of arthropathic changes in the pisotriquetral joint and induce symptoms, such as pain. When symptoms develop, surgical intervention should be considered. Singer et al. treated pisotriquetral joint instability with arthrodesis and achieved a favorable outcome [15], and van Eijzeren et al. treated arthropathic changes in the pisotriquetral joint with pisiform resection and achieved a favorable outcome [16]. There is no consistent viewpoint of treatment, and treatment is performed corresponding to each case.

## 5 Conclusion

Our findings suggest that pisiform subluxation complicating DRF does not influence the short-term clinical outcome, i.e., therapeutic intervention of pisiform subluxation may be unnecessary during treatment of DRF. However, it cannot be ruled out that pisiform instability, which may have developed when DRF occurred, appears as a disorder, such as osteoarthritis of the pisotriquetral joint, a long time after the injury. At present, therapeutic intervention of pisotriquetral joint disorders should be considered when they develop.

## Ethical approval

312, Ethical Approval of Juntendo University Shizuoka Hospital.

## Sources of funding

None.

## Author contribution

YS (first author) mainly make this manuscript and was an assistant of operative procedure. KN (corresponding author) mainly performed medical examinations and surgery for this patient. HO, KM, YI and KK discussed and advised about the treatment for this patient. All authors read and approved the final manuscript.

## Conflicts of interest

All authors have no conflict of interest.

## Research registration number

Researchregistry3430.

## Guarantor

Kiyohito NAITO.
